# The Role of Insulin Within the Socio-Psycho-Biological Framework in Type 2 Diabetes—A Perspective from Psychoneuroimmunology

**DOI:** 10.3390/biomedicines12112539

**Published:** 2024-11-07

**Authors:** Anne Wevers, Silvia San Roman-Mata, Santiago Navarro-Ledesma, Leo Pruimboom

**Affiliations:** 1 Clinical Medicine and Public Health PhD Program, Faculty of Health Sciences, University of Granada, 18071 Granada, Spain; info@anne-wevers.com; 2 Department of Nursing, Faculty of Health Sciences, Campus of Melilla, University of Granada, 52004 Melilla, Spain; silviasanroman@ugr.es; 3 Department of Physical Therapy, Faculty of Health Sciences, Campus of Melilla, University of Granada, 52004 Melilla, Spain; 4 University Chair in Clinical Psychoneuroimmunology, Campus of Melilla, University of Granada and PNI Europe, 52004 Melilla, Spain; leo@cpnieurope.com

**Keywords:** psychoneuroimmunology, psychosocial factors, insulin, mitochondrial information processing system, type 2 diabetes mellitus with musculoskeletal disorders and neurodegeneration, insulin resistance, mitochondrial dynamics, insulin resilience, evolutionary medicine, integrative health interventions

## Abstract

The interplay between socio-psychological factors and biological systems is pivotal in defining human health and disease, particularly in chronic non-communicable diseases. Recent advancements in psychoneuroimmunology and mitochondrial psychobiology have emphasized the significance of psychological factors as critical determinants of disease onset, progression, recurrence, and severity. These insights align with evolutionary biology, psychology, and psychiatry, highlighting the inherent social nature of humans. This study proposes a theory that expands insulin’s role beyond traditional metabolic functions, incorporating it into the Mitochondrial Information Processing System (MIPS) and exploring it from an evolutionary medicine perspective to explore its function in processing psychological and social factors into biological responses. This narrative review comprises data from preclinical animal studies, longitudinal cohort studies, cross-sectional studies, machine learning analyses, and randomized controlled trials, and investigates the role of insulin in health and disease. The result is a proposal for a theoretical framework of insulin as a social substance within the socio-psycho-biological framework, emphasizing its extensive roles in health and disease. Type 2 Diabetes Mellitus (T2DM) with musculoskeletal disorders and neurodegeneration exemplifies this narrative. We suggest further research towards a comprehensive treatment protocol meeting evolutionary expectations, where incorporating psychosocial interventions plays an essential role. By supporting the concept of ‘insulin resilience’ and suggesting the use of heart rate variability to assess insulin resilience, we aim to provide an integrative approach to managing insulin levels and monitoring the effectiveness of interventions. This integrative strategy addresses broader socio-psychological factors, ultimately improving health outcomes for individuals with T2DM and musculoskeletal complications and neurodegeneration while providing new insights into the interplay between socio-psychological factors and biological systems in chronic diseases.

## 1. Introduction

Insulin (INS), traditionally viewed merely as a metabolic regulator implicated in various diseases [1], might hold a more expansive role as a ‘socio-psychological substance’ within the body’s biological systems. This narrative review explores incorporating INS into the Mitochondrial Information Processing System (MIPS), a model that redefines mitochondria not just as cellular powerhouses but as central processors of socio-psychological factors [2]. Such a conceptualization positions mitochondria as ‘social organelles’ essential in processing social and psychological factors through mechanisms of sensing, integration, and transduction—a cornerstone of mitochondrial psychobiology. We hypothesize that INS plays a pivotal role in this process—beyond its established metabolic actions—by cooperating with mitochondria in modulating biological responses to psychological and social factors. We adopt the MIPS [2] model as a theoretical framework for the exploration of INS’s function as a socio-psycho-metabolic hormone with systemic influence.

To further support this hypothesis, we examine INS through an evolutionary lens, considering how its roles may have been shaped by ancient social, psychological and environmental stressors and survival mechanisms. This evolutionary perspective deepens our understanding of INS as a socio-psychological substance and informs the development of interventions that align with human biology’s evolutionary adaptations.

This paradigm shift aims to contextualize INS within a broader socio-psycho-biological framework, enriching our understanding of its multifaceted functions. This narrative review, along with the presented theoretical framework and evolutionary perspective, aims to offer new directions for hypothesis building and establishes a foundation for more systematic experimental approaches. Ultimately, it seeks to contribute to the development of potential new strategies for managing health conditions where INS is a key factor, such as Type 2 Diabetes (T2DM) with associated musculoskeletal (MSK) and neurodegenerative complications.

## 2. Sensing, Integrating and Transducing—A Theoretical Role for Insulin

The MIPS model, as proposed by Picard and Shirihai [2], positions mitochondria as social organelles adept at a three-step process—sensing, integrating, and transducing psychological and social factors into cellular and molecular modifications, next to their classical role at the level of cell- and systemic metabolism. They highlight the dual role of mitochondria as both targets of stress and sources of signaling, essential for biologically embedding both stressful and other psycho-social experiences into our biology [3]. Accumulating evidence in both human and animal studies further underscores the pivotal role of mitochondria in the response to both acute and chronic stress and in the biological embedding of adversity [4]. Their functions are indicated as important mechanisms in understanding how stressful life events ‘get under the skin’ and impact health and physical well-being, recently also shown in studies examining human peripheral tissue [5].

Evolution generated multiple methods of communication between mitochondria and the rest of the cell [6]. The inherent plasticity and dynamic nature of mitochondria are important mitochondrial characteristics and facilitate their roles as processors and communication hubs, allowing them to adapt through functional and morphological changes such as alterations in size, shape, and distribution. These adaptations respond to both internal and external stimuli and are crucial for meeting various demands [7,8]. The adaptive capacity of mitochondria, known as mitochondrial dynamics, specifically involves processes such as fission, fusion, biogenesis, and mitophagy [9]. Mitochondria are pivotal in producing complex signals across biological domains, suggesting their importance as fundamental elements in both cellular and systemic communication networks [10].

Completing the understanding of the MIPS model seems to require the identification of key mediators within this system. Evolutionary pressure has led to the development of multiple communication pathways not only between mitochondria and other organelles [6] within the host cell but also across different cells [11]. Despite recent scientific advances, the construction of a coherent molecular map that fully integrates and delineates mitochondrial functions during psychological stress remains incomplete [12]. Specifically, the molecular mechanisms that control mitochondrial plasticity, dynamics, and their capacity to integrate and respond to a spectrum of cellular, environmental, and developmental stimuli are still to be fully understood [13]. Identifying the biological substrates and pathways that bridge psychological experiences with physiological processes seems important, as these links might be essential for elucidating the mechanisms behind observed biological changes [4]. Given these complexities, a detailed theoretical exploration of potential mediators like INS, and its relevant pathways, as well as elucidating its extensive roles within the MIPS model, seems valuable and relevant.

Insulin resistance (IR) is a central mechanism underlying most chronic diseases [14]. Mitochondrial dysfunction is frequently implicated as a precursor to IR; however, IR itself can also contribute to mitochondrial dysfunction [15]. When viewed through a psychosocial stress model, IR becomes a stronger predictor of the hypothalamus–pituitary–adrenal (HPA) axis response than obesity [16]. This suggests a complex interaction between psychosocial and physiological factors, with INS playing a critical role alongside mitochondria. The proposal to explore INS as a social-psychological substance within MIPS is further based on findings that psychosocial stress acts as an upstream event for IR, with mitochondria as downstream targets. This bidirectional relationship indicates that stress can influence INS sensitivity, which in turn impacts mitochondrial function [17].

The neurons in the arcuate nucleus of the hypothalamus (ARC) are activated by emotional stimuli and express INS receptors [18]. This might further illustrate INS’s role in processing socio-psychological factors. The neurons in the ARC function as master regulators that integrate physiological signals with the energy state of the organism [19]. The melanocortin neurons within this nucleus receive peripheral metabolic information and mediate appropriate behavioral and metabolic responses to maintain energy homeostasis. By signaling energy availability, INS in the ARC coordinates metabolic functions and stress adaptation, reflecting its dual role in both biological and psychosocial processes. INS has also been used as a socio-psycho substance in a historical context, where INS shock therapy was used in psychiatric treatment, highlighting the hormone’s significant impact on the central nervous system. While this practice is no longer in use, it underscores INS’s profound effects on behavior and stress adaptation. This historical use of INS in psychiatric treatment, along with its role in the ARC regulating energy and stress responses, further encourages the exploration of INS as a socio-psychological substance within the MIPS model.

Integrating INS and IR into the MIPS model requires an in-depth understanding of the strong relationship between mitochondrial function and INS and INS receptor sensitivity, as well as an exploration of experimental findings [20]. In this review, we propose a theoretical framework and assess whether it aligns with existing empirical evidence by examining relevant experimental findings. While these findings may support, refine, or challenge the proposed mechanisms, it is important to note that our hypothesis remains speculative, and future research will be required to empirically validate the theory we are constructing.

A recent in vitro study on mouse and human-derived neurons [21], and another study conducting a series of ex vivo and in vivo experiments in obese IR human subjects either with or without T2DM, indicate that INS’s actions directly impact mitochondrial health and efficiency [15,22]. INS plays a role in both the detection of cues and the subsequent adaptations within mitochondria [23]. Mitochondria possess their own genome, and the mitochondrial DNA copy number (mtDNA-CN) serves as a measure of the number of mitochondrial genomes per cell, thus providing possible insights into mitochondrial health [24]. A possible mechanism of INS influence is its ability to affect the regulation of mitochondrial DNA. A study in humans showed associations with INS sensitivity and mtDNA-CN [25]. Mutations and deletions in mtDNA or mitochondrion-related nuclear DNA genes have been indicated in mitochondrial dysfunction [26]. A recent study applying mitochondrial transplantation, in vitro and ex vivo, shows that DNA methyltransferase 1 (DNMT1) translocates to the mitochondrial D-loop region in vascular smooth muscle cells. This hypermethylation represses mitochondrial gene expression, leading to functional damage and reduced mitochondrial respiration [27]. Research in obese human subjects identifies a strong correlation between IR and alterations in methylation feedback loops, indicating that IR possibly leads to mitochondrial alterations through epigenetic mechanisms [28]. IR is strongly associated with a reduction in mtDNA numbers [28].

Often mentioned as a culprit in disease or merely positioned as a metabolic hormone, the above sets the stage to zoom out the microscope and theoretically examine INS’s role as a psychosocial modulator.

Moving forward, the article will explore in greater detail the specific molecular pathways influenced by INS and how these pathways might impact mitochondrial dynamics, subsequently deepening our understanding of how INS might modulate mitochondrial sensing, integration, and transduction of psychosocial factors.

### 2.1. Sensing: The Role of Insulin

To strengthen our proposal of expanding INS’s role beyond metabolism to a broader function as a socio-psycho-metabolic hormone, we will also apply the lens of evolutionary medicine. This approach highlights that, while INS is central to metabolic regulation, it may also play a role in managing stress and social behaviors—both evolutionary pressures that shaped human adaptation. Together, the MIPS model and evolutionary perspective provide a foundation for a theoretical proposal of interventions that consider our evolutionary origins and the psychosocial dimensions involved in conditions where INS is implicated.

The adage ‘nothing in biology makes sense except in the light of evolution’ aptly describes how human physiological responses still mirror those of our ancestors.

Ancient psychosocial stressors are defined as ‘chronic demands that have persisted throughout human evolutionary history’, while modern stressors are described as ‘novel challenges that emerged with the advent of agriculture during the Neolithic period, approximately 10,000 to 12,000 years ago’ [29]. The evolutionary mismatch hypothesis postulates that our behavioral and inflammatory responses, which evolved to help individuals manage ancient stressors (e.g., serious arguments within families or a child confronting frightening situations), are less suited to modern stressors (e.g., commuting to work, hospitalization, family members frequently away, or unemployment) [29].

Psychosocial stressors, whether novel or ancient (e.g., threats from lions), activate deeply ingrained, robust and well-coordinated stress, behavioral and immune responses shaped by natural selection, which likely provided early humans with an evolutionary advantage in dealing with pathogens, predators, and immediate survival threats [30,31,32].

This evolutionary legacy has resulted in an inflammatory bias, which is triggered not only by pathogens but also by psychosocial stressors [32]. These modern psychosocial stressors are recognized as ‘danger signals’, as they pose possible perturbations to homeostasis [18,33].

These non-specific biological responses of the robust IS are mediated by evolutionary conserved neuroendocrine networks of the hypothalamus–pituitary–adrenal (HPA) axis and the sympathetic nervous system (SAM) [34]. Without energy, stress adaptation is not possible [35], highlighting the critical role of these signals and stress axis in increasing the energy demand [30].

Activation of the HPA axis leads to glucocorticoid production such as cortisol (CORT), which in turn increases blood glucose levels through gluconeogenesis, enhancing the availability of energy needed to manage stressors [36]. As blood glucose levels rise, the pancreas detects this increase, and in physiological situations, it triggers the production and release of INS to help regulate and lower the blood glucose levels [37].

The sympathetic–adrenal–medullary (SAM) axis stimulates the release of catecholamines such as dopamine (DOP), norepinephrine (NE), and epinephrine (EPI) [38]. In vitro research has revealed that both human and mouse pancreatic β-cells contain the necessary machinery for catecholamine biosynthesis and signaling. These catecholamines, especially DOP, play a significant role in regulating pancreatic glucagon and INS secretion, showcasing a complex interplay between stress and metabolic regulation [39]. The β-cell stress hypothesis suggests that various factors such as psychological stress can indeed increase INS demand [40].

Physiologic INS secretion follows an oscillating pattern of secretion, marked by distinct periods of ligand and receptor activation [41]. Chronic exposure to INS, but also glucocorticoids (GC) [42], leptin and cytokines [43], triggers a negative feedback loop that over time down-regulates the receptor availability. This creates a state of, in the case of INS, relative hyperinsulinemia. This excess of INS leads to IR due to decreased receptor availability, with catecholamines also hypothesized to be involved in the induction of IR at the receptor level [40,41].

In light of INS’s socio-psychological role, it is important to acknowledge the significant impact of psychological stress, inflammatory reactions and cytokines and chemokines on IR development as well.

In the same way that the stress response activates the cardiovascular, musculoskeletal, and neuroendocrine systems for fight-or-flight, it may also, under certain conditions, prime the immune system to face potential threats posed by the stressor [44].

This involves various mechanisms and mediators, such as the movement and function of dendritic cells, neutrophils, macrophages, and lymphocytes, along with significant local and systemic production of chemokines and cytokines [45].

Various stressors can trigger neuroinflammation responses. An example of immune activation in a psychosocial context is the activation of innate immune receptors known as pattern recognition receptors (PRRs), with the Toll-like receptor (TLR) family being a key example. TLRs are an evolutionary well-conserved family of innate sensors of danger.

These might have their origins as early as the dawn of animal evolution, more than 700 million years ago [33]. These receptors play a crucial role in the immune system by recognizing pathogenic molecules and initiating an immune response. However, research has indicated that TLRs can also be activated by non-infectious stimuli such as stress hormones and metabolic byproducts, which can be elevated in states of psychological stress. Indeed, in response to chronic stress paradigms in rodents, elevated levels of TLR expression in the hippocampus, prefrontal cortex, blood and serum are reported [46]. Exemplifying the latter and the influence of psychosocial factors, studies in macaques have shown low-status-associated polarization of the TLR4 pathway towards a pro-inflammatory response. The latter provides an indication of the (direct) biological effects of social inequity on immune function and the influence of social gradients in health [47]. Another mouse model studying the effects of 4 weeks of social isolation showed an elevated neuroinflammation response, medicated by TLR-4 [48].

The evolutionary theory of loneliness (ETL) posits that social isolation once posed life-threatening risks, leading to the development of robust biological responses [49]. Much like pain signals harm to the body, loneliness signals broken social bonds, triggering stress responses designed to restore connection [39,40]. The lack of social cohesion has also been mentioned in the literature as an evolutionary mismatch [38]. Indicating the contextual importance of the psychosocial environment—and the lack thereof—in health and disease management.

Subsequently, TLRs can stimulate pathways leading to the activation of NF-κB, a transcription factor that regulates the expression of inflammation-related genes, including cytokines. Chronic stress models in mice show a hyperactivation of the NF-κB pathway in brain regions involved in stress response and affect [46]. In rodent models, it has been shown that the TLR-NF-κB pathway is one of the main mechanisms contributing to inflammation in times of chronic stress [46]. The aforementioned mediators prompt both central and peripheral immune cells to release pro-inflammatory cytokines. Cytokines identified in chronic stress paradigms are IL-1β, IL-6, and TNF-α and, to a lesser extent, IL-10, interferon-gamma (IFN-γ), IL-17, IL-22, and IL-4 [46].

**Figure 1:**
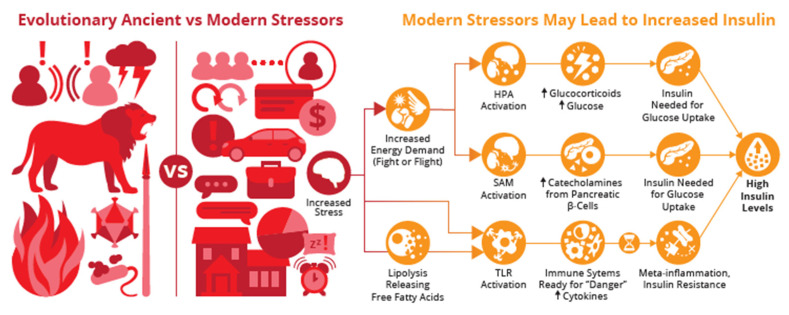
Evolutionary ancient vs. modern stressors and their impact on insulin (INS). Modern stressors activate the HPA and SAM axes, increasing glucocorticoids and catecholamines, leading to elevated glucose and INS demand. Chronic activation induces lipolysis, TLR activation, and meta-inflammation, contributing to hyperinsulinemia and INS resistance (IR).

Whilst stress response mechanisms once enabled survival from environmental threats, modern-day stressors may fail to serve their original function. While adaptive in the short term, prolonged activation of stress mechanisms and cytokine and chemokine production can lead to a pro-inflammatory state, disrupting metabolic processes and inhibiting INS signal transduction processes (see Figure 1) [50].

So-called ‘critical nodes’ form an important part of the signalling network that functions downstream of the INS receptor (INSR) and the INS growth factor-1 receptor (IGF1R). Signaling pathways that are activated by cytokines through receptors such as the TNFR with affinity for tumour necrosis factor-α (TNFα), and the REC receptor, activated by cytokines such as interleukin-6 (IL-6) and leptin, interfere with INS signaling through crosstalk with the critical nodes [51]. Important examples in light of the communication between INS and mitochondria and in the context of this narrative review are the AKT and MAPK pathways discussed in the paragraphs hereafter.

Adipose tissue is part of the stress response as well. Stress, via multiple pathways, induces lipolysis, releasing free fatty acids (FFA) into the circulation. These can directly activate immune cells by TLR receptor activation, leading to further inflammation and cytokine release and influencing the above-described pathways. This is referred to as metainflammation, a portmanteau of metabolism and inflammation [43].

While stress, through the pathways discussed above, can induce IR, conversely, IR and the crosstalk between inflammatory pathways and neurocircuits in the brain can trigger behavioral responses, such as avoidance and heightened alarm [52]. Moreover, dopamine (DOP) and INS signaling systems have been shown to exhibit reciprocal regulatory relationships, emphasizing the interdependence of metabolic and neurochemical processes [53]. Additionally, INS and oxytocin (OXY) share a cross-talk mechanism in glucose homeostasis. OXY which plays a role in the stress response, has been demonstrated to stimulate INS secretion and improve pancreatic function through central regulation via vagal neurons that innervate β-cells [54].

The reciprocal regulation between INS, DOP, and OXY may demonstrate INS’s broader function, beyond its metabolic role, in modulating psychosocial and behavioral processes. This PNI view highlights how INS operates at the intersection of metabolic, immune, neural, and endocrine pathways, emphasizing its relevance in the context of Type 2 Diabetes (T2DM).

Indeed, chronic stress impairs INS signaling (INSS) both in vitro and in vivo in laboratory animals [55], and stressful life events, traumatic experiences, general emotional stress, anger and hostility, distressed sleep, and workplace stress might induce IR [17]. In a longitudinal study with 12,844 Australian women initially free of DMT2, where IR is seen as a hallmark [56], those reporting moderate–high stress levels had a 2.3 times higher risk of developing later T2DM [57].

The psycho-physiological stress response remains one of nature’s fundamental survival mechanisms [45]. While detrimental in the long term, the immune system is simply doing what it is designed to do: protect us [30]. Understanding the latter enables the proposal of theoretical interventions later in this review that support the original function and context of INS and mitochondria.

### 2.2. Sensing: The Role of INS Receptors

The physiological interactions between INS and INS-like growth factor (IGF-1) with cellular receptors such as INS receptors (INSRs), INS-like growth factor 1 receptors (IGF1Rs), and hybrid INS receptors/INS-like growth factor 1 receptors (INSR/IGF1Rs) initiate a cascade of responses that prepare mitochondria to sense and respond to environmental cues effectively, as shown in studies [58,59]. Recent research in mice highlights the significant role of the INS/IGF1 signaling pathways in regulating mitochondrial biogenesis, fusion, architecture, and functionality, underscoring the intricate link between INSS and mitochondrial dynamics [60]. This is setting the stage for an inclusion of INS in the theoretical MIPS model.

In vitro research in Human embryonic Stem Cells (HSCs) shows that when INS binds to its receptors, it triggers a series of signaling cascades, including the AKT-kinase (AKT) and Mitogen-Activated Protein Kinase (MAPK) pathways. Along with their downstream effectors, they facilitate a range of cellular and mitochondrial functions [61]. The AKT pathway predominantly mediates the metabolic effects of INS, whereas the MAPK pathway is crucial for regulating cell growth, proliferation, differentiation, mortality, and survival [62]. It interacts with the AKT pathway and others to modulate cell growth and differentiation [20].

The AKT and MAPK pathways operate in a sequence and context-dependent manner, demonstrating complex cooperation and interaction within cellular signaling networks [63]. Studies, including in rats, indicate that these pathways are not isolated; they work synergistically with each other but also other pathways. They can reciprocally influence each other, where the modification of one pathway can affect the other, either enhancing or inhibiting its functions [8,64,65]. This dynamic interaction is especially crucial in stressed conditions, where external stressors modulate INS levels and signaling, subsequently impacting mitochondrial dynamics [66]. An imbalance in mitochondrial dynamics and dysregulated opposing and counterbalancing processes can cause significant disruptions in mitochondrial function [9]. These disruptions are pivotal as they can precipitate abnormal cellular outcomes and contribute to the development of diseases such as Type 2 Diabetes Mellitus (T2DM), emphasizing the critical implications of INS Signaling (INSS) interplays in disease pathology [9,66]. Depending on their size and morphology, mitochondria exhibit distinct responses to incoming signals. For example, in vitro research shows that larger mitochondria, with a greater matrix volume and a lower surface-area-to-volume ratio, respond differently compared to smaller counterparts [67,68].

### 2.3. Sensing: INS, AKT-Pathway, and Mitochondrial Dynamics

INS and IGF-1 signaling, through their respective receptors, significantly enhance mitochondrial function in a PI3K/AKT-dependent manner, as shown in in vitro studies [69]. Under physiological conditions, INS promotes mitochondrial fusion, while IR triggers mitochondrial fission—the physiological interplay of both processes are crucial for mitochondrial quality control [70]. Furthermore, INS, via the AKT pathway, orchestrates a network of downstream effectors such as Glycogen synthase kinase 3 beta (GSK3β) [71], mammalian target of rapamycin 1 (mTORC1), Forkhead box protein O1 (FoxO1) [68,72], the mammalian target of rapamycin (mTOR), Peroxisome proliferator-activated receptor gamma coactivator 1-alpha (PGC-1α), Unc-51 Like Autophagy Activating Kinase (ULK1), and the pro-apoptotic proteins Bcl-2-associated X protein (BAX) and Bcl-2-associated death promoter (BAD) [72,73]. These effectors have been shown in mice studies [36,37] to collectively influence a range of mitochondrial functions, including biogenesis, mitophagy, fusion, fission, and apoptosis [20,62]. Through its impact on these effectors, INS might facilitate a complex interplay that maintains mitochondrial dynamics [20].

### 2.4. Sensing: INS, MAPK-Pathway and Mitochondrial Dynamics

**Figure 2:**
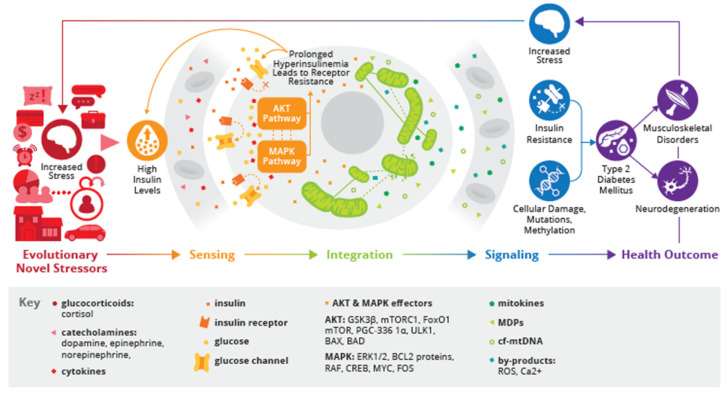
Incorporation of Insulin into the Mitochondrial Information Processing System (MIPS). This figure illustrates the potential role of insulin (INS) within the Mitochondrial Information Processing System (MIPS) in response to psychosocial and evolutionary novel stressors. Using the MIPS model and an evolutionary perspective, we propose that INS functions as a socio-psycho-metabolic hormone that cooperates with mitochondria to mediate the sensing, integration, and signaling of these stressors. This cooperation might affect mitochondrial dynamics through pathways such as AKT and MAPK. Prolonged hyperinsulinemia, driven by chronic stress, may lead to receptor resistance, impacting mitochondrial function. This figure highlights the hypothesized relationship between psychosocial stress, insulin resistance, and the development of complications associated with Type 2 Diabetes Mellitus (T2DM), such as neurodegeneration and musculoskeletal disorders (MSDs). By positioning insulin as a central modulator working in cooperation with mitochondria in the MIPS framework, we propose that it plays a key role in integrating psychosocial and metabolic signals, ultimately influencing health outcomes.

INS modulates the MAPK pathway, which is pivotal for transmitting extracellular signals to cellular responses [20]. The key effectors of this pathway include extracellular signal-regulated kinases 1 and 2 (ERK1/2), which influence mitochondrial functions [74]; BCL2 proteins, involved in apoptosis regulation [74] and Rapidly Accelerated Fibrosarcoma kinase (RAF), as well as transcription factors such as cAMP response element-binding protein (CREB), Myelocytomatosis oncogene (MYC), and FBJ Murine osteosarcoma viral oncogene homolog (FOS), which contribute to mitochondrial biogenesis and mitophagy [20] (see Figure 2).

The above provides theoretical insights into how INS contributes to the sensing of psychosocial factors. However, further clinical studies are necessary to further explore this theory.

#### Insulin’s Role in Sensing: Proof of Concept

In a series of studies involving rats subjected to four distinct models of stress-induced depression—chronic unpredictable mild stress, learned helplessness, chronic restraint, and social defeat—significant modifications were observed in the AKT and MAPK signaling pathways. Notably, phosphorylated AKT (p-AKT) levels were altered across all four depression models, demonstrating the extensive impact of stress on INSS mechanisms [65]. Another study in rats highlighted that social isolation could induce early-stage dysfunctions in the INSS pathways. This was evident through a reduction in AKT phosphorylation, potentially exacerbating an oxidative state and further complicating metabolic homeostasis [75]. The MAPK pathway, critical for cellular processes such as growth and response to stressors, showed significant changes in the context of social defeat stress in rats [76]. As this is a review, the discussed concepts remain theoretical. Further clinical research in humans is necessary to elucidate the role of INS in mitochondrial sensing of psychosocial factors.

### 2.5. Integrating—The Role of Insulin

As part of the sensing function, INS influences cellular dynamics through its receptors, affecting the strength and duration of AKT and MAPK pathway activities. Subsequently, mitochondria integrate these inputs together with information from interactions with other organelles facilitated by mitochondria-associated membranes (MAMs) [2,64]. These structural domains establish physical connections with the nucleus, lysosomes, endoplasmic reticulum (ER), and Golgi apparatus, enhancing cellular coordination and response to changes [77].

This integrative function strengthens our hypothesis that INS might be an important player in the MIPS framework; however, additional research is required.

#### Insulin’s Role in Integration: Proof of Concept

Altered signaling through MAMs is a characteristic feature in various tissues exhibiting IR, such as muscles [78]. This alteration underscores the systemic impact of INS’s regulatory role at the cellular level. A proteomic analysis in rats, which underwent chronic psychological stress, demonstrated significant changes in the MAM proteome [79]. Altogether, the above might serve as a proof of concept that psychological stress, through INSS, influences mitochondrial function. However, it remains theoretical, and further research is needed to confirm these findings.

### 2.6. Transduction—The Role of INS

Mitochondria systematically impact cellular and organismal behaviors through the release of signaling molecules [12]. Mitokines, for example, are released into the systemic circulation and mediate a non-autonomous stress response [24]. This process is suggested to be analogous to bacterial quorum sensing, where signaling molecules coordinate group behaviors in bacterial populations [80]. Similarly, mitokines facilitate intercellular communication, allowing mitochondria to influence distant cells and maintain homeostasis across the organism [81]. This process enhances inter-tissue communication of mitochondrial stress, playing a critical role in synchronizing and amplifying stress responses across the body, thereby maintaining more efficient organismal homeostasis [24]. The increase in mitokines might be an attempt at mitohormesis, enhancing stress resistance [82]. However, the outcomes of mitokine signaling can be both beneficial and detrimental, depending on the signal’s magnitude and duration, indicating that mitokine production has dual aspects [83].

Additionally, the mitochondrial genome (mtDNA) is responsible for producing small mitochondrial DNA (mtDNA)-encoded peptides, such as the mitochondrial open reading frame of the twelve S-c (MOTS-c), and humanin and small HN-like peptides (SHLPs) are classified as mitochondrial-derived peptides (MDPs) [84]. An observational study indicates that INS signaling also influences mitochondrial integrity and immune responses by influencing the release of cell-free mitochondrial DNA (cf-mtDNA) [85]. A recent systematic review of human studies suggest that this cf-mtDNA, integral to mitochondrial damage-associated molecular patterns (mtDAMPs), acts as a potent immunological activator due to the bacterial origin of mitochondria. This activation marks cf-mtDNA as a significant indicator of inflammatory diseases and a predictor of mortality [86,87].

Dysfunctional mitochondria, and altered dynamics, can lead to increased production of reactive oxygen species (ROS) and other byproducts. A recent review from 2024 describes that mitochondrial ROS production can be both a normal function and a sign of dysfunction, depending on the context. In the case of dysfunction, excessive ROS can damage cellular structures, including nuclear DNA, potentially resulting in mutations [88].

Dysfunctional mitochondria also lead to the buildup of, for example, Calcium (Ca^2+^), as per mitochondria’s buffering function [64]. Ca^2+^ levels have quite narrow limits, and small changes can cause negative health outcomes, as will be shown later in the article.

Further to the above, mitochondria generate cellular and organismal behaviors through mitochondrial-nuclear crosstalk and interspecies epigenetic remodeling [12].

Mitochondrial metabolites serve as substrates for epigenetic marks in the nucleus, while others function as mediators that modulate nuclear gene expression. This dynamic interaction results in modifications to both the epigenome and gene expression, which, in turn, regulate mitochondrial function [89]. This systemic communication, possibly influenced by INS as elaborated on hereafter, is essential for regulating gene expression and happens via epigenetic mechanisms such as DNA methylation and histone modification [12,87]. In addition to nuclear DNA (nDNA) methylation, emerging evidence shows that mtDNA can undergo methylation [90]. Methylation of the D-loop region in mtDNA, which regulates both mtDNA replication and transcription, has been observed to increase in individuals with IR. Recent studies in IR obese humans have unveiled, for the first time, an INS signaling-epigenetic-genetic axis that may regulate mitochondria, suggesting that INS has the capability to directly modulate mitochondrial gene expression and mitochondrial function [28]. This may suggest that INS signaling could modulate both nuclear and mitochondrial gene expression, potentially through epigenetic regulation of genes encoded by both mtDNA and nDNA. Data demonstrate impaired mitochondrial structure and function in IR muscle, which may be caused by epigenetic regulation of genes encoded by mtDNA and nuclear DNA [90].

Research on C. elegans has shown perturbations in INSS to possibly lead to changes in mitochondrial function, resulting in significant alterations in gene expression at both transcriptional and translational levels [91]. It is hypothesized that mitochondrial abnormalities may arise from the epigenetic regulation of both mitochondrial and nuclear-encoded genes responsible for mitochondrial structure and function, with INS potentially playing a critical role in modulating these epigenetic changes, particularly through its influence on mitochondrial DNA (mtDNA) methylation and gene expression pathways [92].

Epigenetic modifications are suggested to be key intermediaries in the interactions between environmental stimuli, such as stress, and the genome. These modifications can trigger inflammatory responses that promote metabolic disorders [62].

Ultimately, it might come down to communication. Based on the information above, we hypothesize that INS might act as a socio-psychological communication substance, possibly supporting mitochondria to sense, integrate, and transduce psychosocial signals. This dynamic dialogue between the environment, INS, mitochondria, and the rest of the organism enables maintaining homeostasis, though it might lead to adverse health outcomes if dysregulated. To further validate these findings and hypotheses, additional research involving human subjects is necessary.

#### INS’s Role in Integration: Proof of Concept

Summarizing the above, recent evidence underscores INS’s role in the integration and transduction of psychosocial signals through mitochondrial pathways. Acute mental stress has been shown to elevate levels of circulating mitochondrial DNA (mtDNA), with psychological factors amplifying these effects, suggesting potential mediation by INS. A systematic review of human studies mentions higher cf-mtDNA levels in suicide attempters to correlate with increased CORT response during the dexamethasone suppression test (DST), indicating a possible link between HPA hyperactivity and cf-mtDNA levels [87]. While this suggests an interaction between stress and mitochondrial dynamics, the role of INS remains speculative. Other factors may also contribute to cf-mtDNA release, and further studies are required to explore these potential mechanisms.

Machine learning analyses on human serum suggests that psychological factors, rather than inherent personal traits, predict stress-induced changes in circulating cell-free mtDNA (ccf-mtDNA). Major depression and other conditions linked to psychological stress have been shown to lead to a rise in serum ccf-mtDNA levels [60]. In a study involving 46 healthy middle-aged adults exposed to a brief psychological stressor—a public speaking simulation—a significant surge in serum circulating cell-free mtDNA was documented shortly afterward [93], with potential mediation by INS.

The mechanisms of cf-mtDNA release extend beyond cell death and can also be triggered by psychological stress [94]. A study in 20 healthy young men indeed shows cf-mtDNA release in response to psychosocial stress, suggesting an active release mechanism resulting from psychosocial challenges [95], possibly mediated by INS. Nuancing and further research are important, as variations in cf-mtDNA responses to stressors indicate that not all forms of cf-mtDNA are pro-inflammatory, adding complexity to INS’s role as a social substance affecting physiological responses [56,96]. 

In lower levels of MOTS-c, a mitochondrial-derived peptide, in T2D patients, INS was found to regulate and attenuate the MOTS-c response in a study in humans [54,64,97]. Moreover, conditions like major depression are known to alter mitokine production and cf-mtDNA, as shown in human studies [98].

A recent study performed among older adults indicates that early life stress impacts mitochondrial functionality by altering mtDNA content, affecting cellular respiration and bioenergetics. Adults who suffered childhood maltreatment or experienced parental loss before age 18 typically exhibit reduced mtDNA-CN compared to those without adverse childhood events (ACE) [5].

While the above advances the understanding of INS as a socio-psychological substance and its potential place in the MIPS model, it remains theoretical. Further research is needed to confirm these findings and explore their practical applications.

### 2.7. Type 2 Diabetes Mellitus with Musculoskeletal Problems and Neurodegeneration

Further to the above, we hypothesize that INS acts as a potential key modulator within the MIPS model, orchestrating the interplay between psychosocial risk factors, INS, mitochondrial dynamics, and various health outcomes. Two significant examples that reinforce the role of INS as a social substance and the need for integrative interventions, including those in the psychosocial realm, are Type 2 Diabetes Mellitus (T2DM) with musculoskeletal disorders (MSDs) and T2DM with neurodegeneration.

In the sections that follow, we will explore how the models discussed earlier could theoretically contribute to the knowledge about the development of T2DM with MSDs and neurodegeneration. We will highlight the role of psychosocial factors in shaping health and disease, consider INS’s role as a ‘social substance’ within the MIPS model, and illustrate how the function of the MS system might extend beyond mere movement, potentially acting—under the influence of INS and mitochondria—as a component in the psychosocial-biological axis or as a psychobiological interface. To better understand this interplay, we look at recent research and studies that demonstrate the effects of psychosocial stressors, INS and mitochondrial function in T2DM and subsequent musculoskeletal- and neurological health and complications.

#### 2.7.1. Type 2 Diabetes Mellitus with Musculoskeletal Disorders

Studies in rodent models have demonstrated that adverse childhood experiences (ACE) lead to altered mitochondrial structure and bioenergetics in peripheral muscle. Duchowny’s research revealed compromised maximal ATP production in skeletal muscle (SM) mitochondria among individuals who experienced ACEs, with each additional adverse event associated with modestly lower ATPmax, a measure of maximal capacity for ATP production [5]. A systematic review and meta-analysis from 2024 on human cohort studies indicates that Musculoskeletal Disorders (MSDs) are prevalent in individuals with T2DM [99]. There seems to exist a vicious cycle where T2DM and MSDs mutually exacerbate each other. This interconnection is largely driven by skeletal muscle (SM) IR and mitochondrial dysfunction, which are identified as the key pathophysiological links between T2DM and MSDs [100]. A significant reduction in mtDNA-CN has been documented in critical tissues such as skeletal muscle (SM), adipose tissue, and peripheral blood in individuals with obesity and T2DM. A population-based follow-up study suggests that this decrease in mtDNA-CN often precedes the clinical onset of T2DM, which might show its potential as an early biomarker for disease prediction and a target for therapeutic intervention [28,101].

IR can lead to mitochondrial dysfunction, which in turn results in hyperglycemia and its metabolic consequences [102]. This hyperglycemia contributes to the glycosylation of collagen structures, reducing bone flexibility. Advanced glycation end-products (AGEs) accumulate in musculoskeletal (MSK) tissues, adversely affecting biomechanical properties by altering charges and forming collagen cross-linkages. Such mechanisms are linked to various T2DM-related MSD, including osteoporosis, osteoarthritis, sarcopenia, tendinopathy, neuropathy, and joint stiffness [103]. Additionally, AGEs induce structural changes in myofibrillar proteins of muscles, further impacting the MS system [100]. Integrating psychosocial factors into this narrative, recent studies in adult mice exposed to social isolation for four weeks showed significant reductions in bone quality, including reduced bone mineral density [104]. This indicates an impact of psychosocial stressors on MS health, further highlighting the intricate interplay between INS, psychosocial factors, and the musculoskeletal system.

Under conditions of glucose overload, mitochondria experience an increase in ROS production and oxidative stress (OS), which can significantly reduce ATP production [100]. In the context of hyperglycemia, mitochondrial fragmentation precedes ROS production induced by high glucose levels. Hyperglycemia increases ROS production within approximately 30 min, and fragmented mitochondria produce about 50% more ROS compared to their filamentous counterparts in the same cell [2]. This might suggest an important role of mitochondrial dynamics in ROS production and suggests the potential effects of psychosocial factors and INS on mitochondrial function and subsequent MSD.

ROS can both activate and repress the nuclear factor kappa-light-chain-enhancer of activated B cells (NF-κB), signaling in a phase- and context-dependent manner [105]. This factor plays a pivotal role in driving the increased expression of inflammatory cytokines and chemokines, exacerbating inflammation [106]. This inflammatory response further contributes to the cycle earlier described and MS deterioration seen in T2DM. Muscle cells, with their relatively high-energy demands, possess a notably high concentration of mitochondria to meet these demands [107]. As such, IR and impaired glucose uptake, but also more chances of high ROS levels in SM, contribute to a spectrum of adverse conditions, including mitochondrial dysfunction [100]. Indeed, in the SM of individuals with IR or T2DM, mitochondria tend to be smaller and fewer [20,108]. This mitochondrial insufficiency in SM correlates with decreased muscle performance, reduced mobility, and increased susceptibility to frailty and muscle atrophy [5].

Additionally, a recent review from 2024 reports elevated production of lipid intermediates such as diacylglycerol (DAG) and ceramides, as well as pro-inflammatory cytokines resulting from intramyocellular lipid deposition. Moreover, the myomitokine GDF15 is extensively studied as a biomarker for various diseases, including those affecting the MSS, further emphasizing the wide-ranging impacts of IR, mitochondrial dysfunction and its subsequent transduction substances such as mitokines [83].

Following the cascade of IR and mitochondrial dysfunction, mitochondrial abnormalities significantly influence muscle and multisystem diseases through various interconnected mechanisms. Mitochondrial defects can lead to muscle diseases due to a loss of oxidative phosphorylation (OXPHOS), abnormalities in mitochondrial dynamics, atypical mitochondrial structures, and morphologies, disrupted energy metabolism, mitophagy, and apoptosis [109]. Specifically, certain mitochondrial mutations or dysfunctions have been identified and as triggers for muscular dystrophy [106]. The literature describes that in SM fibers, mitochondria are strategically positioned between myofibrils, encircle the nucleus, and are densely packed within the sarcolemma [110]. Dysfunction often originates from the loss of structural integrity during Mitochondrial Outer Membrane Permeabilization (MOMP) and Mitochondrial Inner Membrane Permeabilization (MIMP), which are typically triggered by the activation of BAX and BAK proteins [111]—earlier elucidated as effectors of INS pathways. Such events lead to the release of mitochondrial byproducts such as cytochrome c (CYTC), apoptosis-inducing factor (AIF), caspase-9, and Ca^2+^ into the cytosol, thereby initiating programmed cell death pathways [106]. Mitochondrial defects or abnormalities can precipitate muscle and multisystem diseases through various mechanisms, notably including dysregulated Ca^2+^ signaling [106]. SM mitochondria play a crucial role in the storage and regulation of Ca^2+^, essential for maintaining cellular functions [107]. Marchioretti and colleagues show in their study on skeletal muscle of mice and patients that dysfunctions such as excitation–contraction coupling (ECC) deregulation and impaired mitochondrial respiration often precede shifts in muscle force, Ca^2+^ imbalances, and the breakdown of triad structures. Ca^2+^ overload, resulting from leakage from the sarcoplasmic reticulum (SR), impaired Ca^2+^ reuptake into the SR, or increased Ca^2+^ influx through the cell membrane, can activate Ca^2+^-dependent proteins such as calpains, which then degrade intracellular proteins, cellular membranes, and nuclear DNA [112]. A study in mice demonstrated that intramuscular injections of allogenic mitochondria from healthy animals into the hind limbs of mdx mice alleviated SM damage and reduced Ca^2+^ deposits [113]. Additionally, high glucocorticoid doses, often associated with stress and subsequent IR, are known to influence mitochondria in a biphasic manner with high glucocorticoid doses reducing Ca^2+^ buffering capacity, increase ROS production, and heighten susceptibility to cell death [35], which is confirmed as well in an in vitro study [114]. Research further shows that IR and T2DM are associated with dysregulated Ca^2+^ homeostasis, with higher serum Ca^2+^ levels correlating positively with fasting blood glucose and the IR index in humans [115]. The regulation of mitochondrial Ca^2+^ is particularly significant in excitable cells such as skeletal and smooth muscle, where insufficient mitochondrial Ca^2+^ uptake can impair cell function, while Ca^2+^ overload may lead to cellular injury and apoptosis [116]. Notably, an in vitro study showed that, compared to smaller fragmented mitochondria, larger tubular mitochondria in the same cell exhibit similar Ca^2+^ uptake rates but recover more quickly, highlighting how mitochondrial dynamics influence their capacity to modulate Ca^2+^ levels and buffering [117].

Furthermore, macrophages within SM exhibit significant phenotypic diversity, adapting their behavior by switching between M1 and M2 phenotypes in response to environmental changes [118]. A recent review explains that macrophages are not only crucial for tissue homeostasis and the response to injury but are also linked to IR in SM. The concentration of macrophages in SM is intricately linked to INS sensitivity; a negative correlation exists, where higher macrophage presence often signifies lower INS sensitivity [119]. Mitochondrial metabolic alterations significantly influence gene expression, leading to varied responses in immune cells. Specifically, M1 macrophages, which are compromised in their ability to complete the tricarboxylic acid (TCA) cycle, tend to adopt a pro-inflammatory state. In contrast, M2 macrophages engage in β-oxidation, exhibiting anti-inflammatory responses, highlighting their role in maintaining homeostasis and promoting healing [120,121]. During the healing of tendon injuries in mice, tendons of T2DM mice exhibit an increased expression of markers associated with pro-inflammatory M1 macrophages, alongside a prolonged and elevated expression of markers for anti-inflammatory M2 macrophages, which are involved in the decomposition of the extracellular matrix (ECM) [122]. The dysfunction of mitochondria can lead to an accumulation of metabolites, which in turn promotes a shift toward pro-inflammatory M1 phenotypes. This shift is pivotal in the context of SM IR, where macrophages play a significant role [118].

Additionally, recent studies in humans have identified a link between mitochondrial dysfunction and INS sensitivity in conditions like fibromyalgia (FM), indicating that similar INS and mitochondrial impairments could be underlying factors in various MS and metabolic disorders [123]. A review summarizing studies in animals as well as a few in humans describes that hyperglycemia-induced aberrant levels of INS or INS growth factors (IGF) may lead to neuropathic complications, intensifying pain through mechanisms such as central sensitization. Commonly, neuropathic joints manifest in the foot and ankle of patients, and conditions like diabetic polyneuropathy and rheumatoid arthritis (RA)-associated pain are complications [124]. Oxidative stress, driven by elevated ROS and prolonged hyperglycemia in T2DM, is known to contribute to nerve damage, which plays a role in the development of diabetic neuropathy. The latter might manifest as pain [125]. Alternatively, these effects can be mediated indirectly through mitochondrial damage and heightened inflammation [126]. This dynamic might ultimately illustrate how psychosocial ‘pain’, via INS and mitochondria, can exacerbate physical pain and possibly lead to additional complications at the physiological level.

#### 2.7.2. Type 2 Diabetes Mellitus with Neurodegeneration

People suffering from Type 2 Diabetes Mellitus (T2DM) often experience a cluster of symptoms, including pain, chronic fatigue, depression, and circadian disruptions [127]. Besides the role of the musculoskeletal (MSK) system, these health issues likely share another common factor: hippocampal atrophy [128]. The hippocampus is particularly vulnerable to uncontrollable stress [129]. Research in rats has shown for the first time that social defeat exposure, psychosocial threat of attack, and severe psychological stress are combined with divergent structural remodeling of dendrites in hippocampal neurons [130]. Furthermore, another study in rats indicated that the rhythmic expression of the hippocampal transcriptome, as well as the circadian regulation of synaptic plasticity, was misaligned with natural light/dark circadian-entraining [131].

A human study showed changes in the structure, perfusion, and function of the hippocampus in T2DM [132]. Moreover, IR-induced abnormalities in the hippocampus of obese/T2DM patients include inflammatory stress, oxidative stress, mitochondrial stress, and increased generation of advanced glycated end products [133]. Meanwhile, the hippocampus is particularly vulnerable to uncontrollable stress [129], such as emotional, cognitive, social, and metabolic stress, showing another example of how T2DM and its diverse set of complications could be seen in light of the socio-psycho-biological framework.

Altogether, SM exhibits remarkable adaptability to changing metabolic demands, a characteristic that extends to its response to psychological stressors [134]. Indeed, emerging research in human peripheral tissue supports a hypothesis that mitochondria within SM might be integrators of these adverse experiences, effectively linking metabolic responses with broader social and environmental factors [5]. This adaptability can be explained by looking at muscles, the brain, INS and psychosocial factors, from an evolutionary point of view, as we will attempt hereafter.

Under normal physiological conditions, SM is responsible for approximately 80% of postprandial glucose uptake [135]. However, in the presence of IR, this process is inhibited as the IS prioritizes its own energy needs, described as acting ‘selfishly’ [136]. This shift in glucose allocation might be a strategic response by the IS to harness more resources during periods of stress, reflecting an evolutionary mechanism designed to enhance survival. Consequently, IR might redirect glucose away from SMs towards immune functions, supporting the body’s immediate defense mechanisms but leading to reduced glucose availability for muscle activity. For this reason, muscle has been referred to as the ‘forgotten’ organ of the IS [137]. This adaptation, while beneficial in short-term immune responses, might lead to chronic MSDs and chronic pain if the IS remains continuously activated without actual threats, as is often the case with modern psychological stressors [138]. The same goes for the brain. While the brain and immune system might be the only two systems that can dominate all others by extracting resources, including glucose, the selfish immune system has been hypothesized to have the capacity to override the selfish brain. The adagio ‘prima vivere e dopo filosofare’ (first live and then philosophize) summarizes this situation well [30]. In times of stress, inhibiting the hippocampus’s function of regulating the hypothalamus–pituitary–adrenal (HPA) axis might be beneficial. This inhibition facilitates the release of energy-demanding substances necessary for an immediate stress response. However, when the stressor becomes chronic, as is often the case with modern life stressors, prolonged activation of the HPA axis can have detrimental effects. One significant consequence is hippocampal atrophy, which might impair cognitive functions and overall brain health [139].

Although clinical studies are necessary and the jury remains out, the above emphasizes the importance of further investigating the psychosocial factors in the treatment of type 2 diabetes mellitus (T2DM) and the relevance of taking its interrelated evolutionary biology into account while designing prevention as well as treatment protocols for T2DM and other INS implicated conditions. It underscores the need for further research towards an integrative treatment approach in T2DM and highlights the importance of future research to deepen our understanding of the evolutionary-determined socio-psycho-biological cascade. This includes examining the interconnected mechanisms of psychological factors, insulin resistance, mitochondrial dysfunction, and T2DM with musculoskeletal- and/or neurological complications, as well as the role of evolutionary expectations. The latter will be further elaborated on in the following section.

## 3. Discussion: Zooming Out the Microscope on INS-Implicated Diseases and Treatments

This article has explored the socio-psycho-biological framework and INS’s potential place herein. Hereafter, we will theoretically explore interventions and possible parameters that might have an impact at different layers in the proposed socio-psycho-biological framework, and specifically INS, considering its evolutionary origins, enabling the exploration of an integrative approach towards mechanisms involved in T2DM with MSD and neurodegeneration. It needs to be highlighted that it remains a theoretical exploration; further studies are needed to validate the theoretical framework proposed throughout this article and the following paragraphs.

### 3.1. Expectations of Evolution

Evolutionary medicine suggests that many chronic diseases might stem from a mismatch between our evolved bodies and modern human-induced environments [140]. This theoretical framework, combined with the previously proposed model of insulin (INS) as a social substance within the MIPS, is used to explore interventions at multiple levels of this model, as well as methods for measuring their effectiveness, altogether providing a theoretical yet comprehensive overview to guide future research.

Within the evolutionary framework, we assume the existence of ‘expectations of evolution’, which we define as the set of conditions, behaviors, and environmental factors that human bodies have evolved to thrive under, based on the selective pressures experienced by our ancestors. By aligning interventions with these ‘evolutionary expectations’, we might potentially enhance health outcomes by addressing the root causes of these mismatches.

Within the definition ‘expectations of evolution’ we distinguish between evolutionary stressors and evolutionary buffers. Interventions based on stressors are challenges that required adaptation for survival and often had beneficial effects on health through hormesis. Evolutionary buffers, on the other hand, are natural factors that were consistently present in the environment and are essential for optimal functioning and health.

It is important to note that the applications of the theory of evolutionary mismatch might have limitations and should be used with caution, considering that ancestral conditions should not be oversimplified [141]. Careful monitoring and scientific research are necessary to address these concerns and prove the use of this theoretical framework.

### 3.2. Evolutionary Stressors

We propose resilience to embody a system’s capacity to adjust to alterations, encompassing both tolerance and adaptability to a spectrum of stresses—spanning physical, chemical, biological, and psychological domains. Mitochondria, pivotal entities in both health and disease paradigms, emerge as principal focal points in hormetic methodologies, commonly referred to as mitohormesis [142]. These strategies aim to fortify mitochondrial resilience, fostering what has been termed mitoresilience [143]. Hormesis is characterized by a biphasic dose-response to specific mild stressors, such as fasting, intake of polyphenols, exercising, physical and chemical stress, and mental engagement [143]. These stimuli elicit beneficial cellular metabolic pathways influenced by INS signaling (INS), such as the down-regulation of mTOR and IGF-1 [143].

Coping with ‘ancient mild stress factors’—including intermittent fasting, exposure to diverse environmental stresses like cold, heat, hypoxia, and hypercapnia, alongside strategies such as calorie restriction and time-based food intake—has shown significant impacts on various mitochondrial parameters and INS levels [64,144]. This has been demonstrated in a study on human subjects who followed a 10-day protocol including a combination of ‘ancient stress factors’ and hermetic interventions. This study showed an improvement in anthropometrics and metabolic indices, including INS [145].

In the context of T2DM with metabolic syndrome (MS) and hippocampal atrophy, aerobic training has been shown in a clinical trial of 100 patients with T2DM to increase total and right hippocampal volume and protect cognitive function [146]. Exercise also induces adaptations in the MSS, which is beneficial in the prevention and treatment of T2DM [147]. Implementing a ‘ketogenic status’, intermittently steering the system in and out of ketosis, has also demonstrated benefits. Recent studies have shown a ketogenic diet to improve muscle mass and function in mice [148] and influence brain gene expression involved in neurodegenerative disease in T2DM mice [149]. An intervention study in nine patients with T2DM showed that cold acclimatization improves skeletal muscle insulin sensitivity by 43%, comparable to the effects of exercise training [150]. In a recent study in mice, cold exposure was shown to affect the brain peptidome and gut microbiome [151]. A study involving 14 patients with T2DM showed that therapeutic intermittent hypoxia influences multiple mechanisms in T2DM, including blood glucose regulation [152]. Although nutritional interventions have been extensively studied, breath work and temperature interventions, with their diverse forms and mechanisms, remain less systematically explored. Their specific effects on T2DM with MSD and neurodegeneration warrant further research.

It is important to mention there are critics of hormesis and subsequent interventions as well. There seems to be controversy on the interpretation and a lack of standardization, such as the definition and application of hormesis, leading to inconsistent use across different studies and contexts. There seems to be individual variability of the responses to doses, the biological mechanisms are not yet fully understood, and there is a dependence on the context, making it difficult to generalize findings [153]. To rectify the uncertainties, further research is needed in both healthy and specific populations such as T2DM patients, and careful monitoring with tools (such as HRV), which are discussed later, could improve these uncertainties.

### 3.3. Evolutionary Buffers

In addition to stressors, there is another category we propose to introduce as ‘evolutionary buffers’—factors that have been consistently present during evolution. An example is varied food intake, which has been shown to improve INS sensitivity and mitochondrial health.

The plant diversity of homo sapiens’ early diet was composed of over 3000 species [144]. Low nutrient variety is apparent in modern life where people eat an average of twenty ingredients or fewer, which is associated with several chronic diseases, including T2DM. Perhaps the most intruding factor of food variety is provided when the food is fermented [144]. Indeed, a study including in vitro and in vivo experiments in a variety of human cell types shows the activation of Nrf2 cell defense pathways by ancient foods, including fermented vegetables [154], as well as fermented vegetables and spices as NRF2-induced enzyme agonists [64] influencing mitochondria. Another literature study summarizes in vitro and in vivo and clinical studies about the antidiabetic properties of fermented foods [155]. Another case-control study has shown that a higher dietary diversity score might diminish the odds of complications in T2DM [156].

Another evolutionary support might be the maintenance of a healthy circadian rhythm. The circadian timing system includes a central clock in the suprachiasmatic nucleus (SCN) and tissue-specific clocks in peripheral tissues [157]. Desynchronization between the central and peripheral clocks is linked to a higher incidence of insulin resistance (IR) and related diseases [158]. A cross-sectional study in patients indicated that disruptions in the circadian clock may be linked to the disruption of several mechanisms and conditions mentioned earlier, including desynchronization of the SNS and the HPA-axis, IR but also mitochondrial dysfunction and chronic musculoskeletal pain and hippocampal atrophy [159]. Evidence from experimental animal studies as well as controlled human subjects has shown that sleep deprivation and circadian misalignment can both directly drive metabolic dysfunction, possibly causing diabetes [160]. A recent review summarizes that sleep interventions such as sleep education and cognitive therapy have been shown to improve glucose homeostasis [161]. Also, stimulus control, sleep restriction, relaxation and sleep hygiene, as well as bright light therapy and timed melatonin administration are currently being studied with a focus on their effects on glucose metabolism [162]. While there are studies showing the benefits of sleep interventions in T2DM, research is still in its infancy and more research is necessary [163]. A newly proposed circadian rhythm-based intervention might be photobiomodulation (PBM). A recent randomized controlled trial has shown the impact of photobiomodulation of circadian variation in blood pressure, pain pressure threshold and elasticity of tissue as well as improvement in musculoskeletal and neuropathic pain in fibromyalgia (FM) patients [159], a condition in which IR is thought to play a role, with FM being a common comorbidity in individuals with T2DM [164].

A recent systematic review describes that the hippocampus has a unique metabolism requiring high energy for optimal function, especially in humans. PBM is proposed to upregulate mitochondrial activity. It also reports the improvement of self-efficacy and pain catastrophizing, as well as psychological factors [165]. Thereby, PBM, including transcranial PBM, has been suggested to enhance neuronal bioenergetics, cerebral blood flow, oxidative stress, neuroinflammation, neural cell survival, neurotrophic factors, neurogenesis, and brain circuit functions [166]. A study in sleep-deprived mice shows improvement of hippocampal function by the use of PBM [167]. This, in turn, regulates the central nervous system and, subsequently, the peripheral nervous system, which via the earlier discussed cascade could improve central and peripheral sensitization symptoms in individuals with DMT2 with MSD and chronic pain [168]. An RCT, in 34 patients with major depressive disorder, showed that using hyperthermia (WBH) in individuals with depression might be effective [169]. WBH improves IR as well [170]. Similarly, whole-body PBM has recently shown promising results in an RCT, reducing pain and improving quality of life, as well as psychological factors like kinesiophobia, pain catastrophizing, and self-efficacy in patients with fibromyalgia [171]. The proposed underlying mechanisms in T2DM might be similar, ultimately promoting hippocampal neurogenesis; however, further research is necessary to confirm this hypothesis. PBM, thus, could be a valuable treatment modality, emerging as a promising multifactorial intervention [171] spanning a major part of the extended MIPS model and fitting into the evolutionary framework as per the evolutionary mechanisms of photon–biological tissue interactions. As it is improving psychological factors, as well as multiple mechanisms in the previously proposed model, it is therefore a valuable option in the treatment of T2DM with MSD. It also shows the potential of modern technology being able to fulfill evolutionary expectations.

As discussed earlier, human physiological responses still mirror those of our ancestors, even though modern ‘threats’ like academic exams do not require physical action.

Indeed, a meta-analysis shows that these threats can lead to physical consequences as if the stressor should be solved by movement [172]. This might suggest that movement could serve as a buffer, aligning with the system’s evolutionary design to release built-up energy after a threat or as a signal to move away to avoid becoming prey [173]. Although humans evolved for regular physical activity, they were also selected to avoid unnecessary exertion due to energy limitations in early environments. This creates a paradox: despite the known health benefits of exercise, people tend to avoid it unless it is either necessary or enjoyable. To overcome this inertia, but also to facilitate movement as an intervention in conditions where INS is implicated, nudging and altering environments might be of importance, making it more integrated into daily life as it was for our ancestors [173].

Given the integrative nature of the socio-psycho-biological framework, the focus of this study and discussing evolutionary expectations, the recognition of the role of psychosocial factors and subsequent interventions in shaping glycemic control and overall health may be the most important.

A systematic review, as well as a systematic review and meta-analysis, demonstrated the effectiveness of mindfulness-based interventions (MBI) in enhancing glycemic control for individuals with T2DM [174,175]. These interventions, which include mindfulness-based stress reduction (MBSR) and mindfulness-based cognitive therapy (MBCT), not only improve glycemic control but also enhance psychological well-being, illustrating the profound interaction between psychological health and metabolic regulation mediated by INS as a social substance [175]. As the included studies sometimes lack methodological quality, further research is necessary to elucidate the effects of MBI.

Similarly, yoga might support autonomic balance, vagal modulation, hormonal regulation, and glycemic control through mechanisms that involve neurophysiological, neuroendocrinological, and psychophysiological systems [176]. These systems are intricately connected with INS’s role in cellular signaling and energy metabolism, further emphasizing the substance’s broad impact on health beyond its traditional metabolic functions [176].

Additionally, an RCT on Baduanjin, a form of Qigong, shows promising results in preventing and improving hyperlipidemia, primarily by promoting glucose decomposition and consumption. This practice might not only improve the psychological state but also relieve anxiety and depression, spread knowledge of health maintenance, and change dietary customs, demonstrating the possible wide-reaching effects of lifestyle interventions on INS-related pathways [177].

Mindful exercise programs such as Tai Chi, Qigong, and yoga might provide long-term benefits for chronic pain management, particularly in conditions like fibromyalgia [177,178]. They offer pain relief while improving glycemic control.

A randomized controlled pilot study suggests that Qigong may serve as a possible complementary therapy for individuals with T2DM, providing a holistic approach to managing this condition through INS modulation and psychosocial improvements [179].

A prospective study in 100 patients with T2DM shows that diaphragmatic breathing and systematic relaxation techniques, when practiced consistently, can amplify the effectiveness of conventional medication and enhance glycemic control and mental health in T2DM patients [180]. By influencing the autonomic nervous system, these practices optimize heart rate variability (HRV) and reduce diabetes distress.

A prospective mediation study in 440 diabetes patients (both type 1 and type 2) showed that meta-cognitive beliefs (such as ‘worrying about the future keeps me prepared’ or ‘worry is uncontrollable’) are associated with anxiety as well as depression and T2DM. Metacognitive beliefs predict rumination and psychological distress independently of illness representations in adults with T2DM [181]. This suggests that metacognitive therapy might be an effective complementary intervention.

The literature describes that faith-based interventions (FBIs) involve leveraging personal resources like knowledge, self-efficacy, spiritual beliefs, and symptom management skills to promote self-management attitudes (dieting, exercising, emotion regulation) and integrate health with spirituality. FBIs might provide an important framework for enhancing T2DM management through increased social support, strengthened spiritual beliefs and cognition, and improved emotion regulation [182].

Furthermore, psychoeducation about emotions and their evolutionary significance might be an interesting avenue to explore, addressing the psycho-socio-biological framework for conditions where INS is implicated. Emotions like fear, stress and loneliness trigger physiological and behavioral responses designed to protect and guide the organism [183]. By understanding emotions from an evolutionary standpoint, interventions can be aligned with our biological and psychological needs—an example being loneliness.

Early in human history, survival and prosperity depended on banding together—in couples, families, or tribes—for mutual protection and assistance. The pain of loneliness served as a prompt to renew social connections necessary for survival, fostering social trust, cohesiveness, and collective action. Although loneliness can feel like a negative emotion without value, it may have evolved as an aversive state, similar to hunger or pain, to drive behavior change [184]. This understanding of emotional drivers—of which loneliness is one of multiple examples—can offer actionable insights for individuals managing INS-implicated diseases, as psychoeducation empowers people to recognize emotional states and make informed decisions. This, in turn, might support immune function and overall health [184]. This can inform as well on the need of mindfulness interventions or support networks and social interventions, which will be discussed hereafter.

Lastly, it has been suggested for decades that social networks are causally related to disease and mortality risk, and tribe/clan interventions have been recognized as important in the improvement of mitochondrial health [64]. Social interactions are fundamental human needs, and the literature suggests that humans have evolved the need for social connection [185]. Ignoring these evolutionary expectations might impair the IS and diminish INS’s efficacy. However, this field of research and its potential for implementation into T2DM care is still in its infancy. Research indicates that social networks play a crucial role in the prevention of T2DM. Social ties significantly influence individuals’ perceptions, behaviors, and norms related to health behaviors, highlighting the importance of incorporating social networks into lifestyle interventions and T2DM management [186]. While this field is emerging, there is a notable need for more detailed studies that assess the specific benefits of integrating social networks into health interventions. However, as discussed earlier, there is strong longitudinal evidence that social isolation, particularly in men, and a general lack of social support in both genders are associated with an increased risk of developing T2DM [187]. The literature suggests that insufficient social support is linked to a heightened risk of severe T2D complications and that social support can significantly enhance T2DM self-management efforts [186].

Social support might serve as a crucial buffer in T2DM, promoting glycemic control. Social network intervention showed improved integration of patients within their existing networks, leading to a greater reduction in HbA1c and blood glucose, as well as improved behavior-mediating outcomes, as described in a prospective observational study [188].

We propose that in an environment with evolutionary expectations, such as acute stressors and evolutionary buffers proposed in this study, even individuals carrying genes that contribute to diabetes when food is plentiful and sedentary lifestyles are common, are less likely to develop DMT2. However, further studies need to be carried out to prove this hypothesis.

As an outcome measure, building upon the concept of mitoresilience previously discussed [143], we propose introducing ‘insulin resilience’ as an additional dimension within the psycho-social-biological framework aimed at addressing insulin-related conditions. This concept shifts the perception of INS from being merely a culprit in disease to a beneficial player in health management. By focusing on INS resilience, we could improve health communication with health-enhancing messages that align with the theory of regulatory focus [189].

The term ‘INS resilience’ aligns well with proposed interventions designed to be health-promoting rather than merely treating disease or IR. However, it is important to note the limitations of regulatory focus theory, particularly its complexity in real clinical settings and its relevance in the specific context of patients with T2DM [190]. Therefore, the use of the term ‘INS resilience’ requires further research to fully understand its applicability and impact in managing T2DM.

### 3.4. Complementary Tools to Measure the Impact of Interventions for Insulin Resilience

Furthermore, we advocate for the incorporation of Heart Rate Variability (HRV), previously proposed as a parameter for mitoresilience and individual resilience, into this comprehensive approach [143]. IR might be a hallmark of T2DM [56]. The literature states IR is closely linked to the performance of the autonomic nervous system (ANS) [191]. A systematic review and meta-analysis mentioned that IR is closely associated with a heightened sympathetic tone or vagal imbalance and reduced HRV values [192]. This autonomic impairment might be due to hyperinsulinemia alone or with hyperglycemia, potentially damaging peripheral nerves [193]. An observational study on overweight individuals with IR exhibited increased central activation of the HPA during hypoglycemia and showed that IR is a stronger predictor of the HPA response than obesity [16]. This suggests that factors beyond the classical nutritional risk factors might play a role in IR, which could support the hypothesis proposed in this article of INS functioning as—amongst others—a social substance. HRV has shown a correlation with HbA1c levels, suggesting its potential as a possible metric for monitoring glycemic control [194].

A meta-analysis and review of HRV suggests that the current neurobiological evidence informs HRV to be impacted by stress and supports its use for the objective assessment of psychological health and stress [195]. A recent observational study on 197 participants indicated that RSI—an HRV-based stress index—reacts to physiological changes related to psychosocial stress and recovery [16,196]. HRV has emerged as an important tool to measure the physiological response to stress, both in adults and children, as well as in research fields of psychiatric conditions and biological psychology [197].

Lastly, there might be a connection between HRV and mitochondria, as explained in a recent review from 2024. A bidirectional relationship seems to exist between the ANS and mitochondria where autonomic activity is thought to directly impact mitochondrial function, while mitochondria participate in and modulate ANS function and signaling [198]. Examples given are co-enzyme Q—a mitochondrial enzyme—enhancing ANS function in healthy humans [199], while mitochondria-derived ROS might mediate sympathetic activity [200]. Thereby, one of the first studies of a potential autonomic mechanism directly linking stress to mitochondrial dysfunction, which was a study in rats, showed that chronic stress results in persistent sympathetically mediated effects that subsequently alter mitochondrial function [201].

There are limitations on the use of HRV, and to the best of our knowledge, there is currently no consensus on normal ranges for these parameters, neither for healthy individuals nor for T2DM patients [197]. Furthermore, HRV is influenced by factors beyond stress and autonomic function, making it challenging to isolate the impact of single factors. For these reasons, the use of HRV in the specific context of this article requires further research.

Given the heterogeneity in the etiology of T2DM [56] and the subsequent need for personalized interventions and measurement tools, HRV emerges as a promising, affordable, and non-invasive tool. HRV shows sensitivity to psychosocial factors as well as T2DM hallmarks and parameters related to mitochondrial health, making it a valuable and complementary addition to existing clinical practices. Unlike parameters such as HbA1c and the Homeostatic Model Assessment (HOMA) index, which require professional evaluation and may limit patients’ self-management capabilities, HRV allows for regular self-monitoring. This enables patients to actively participate in their health management, facilitating more frequent measurements and tracking the impact of various interventions on their condition.

It might also allow validation of the theoretical framework and subsequent interventions proposed in this article, given its sensitivity to IR, hyperglycemia, mitochondrial health, and psychological stress factors. This comprehensive sensitivity supports measuring the effects of personalized treatment methods, helping to detect trends and assess the effectiveness of specific interventions for individual patients. This approach is especially beneficial for patients seeking to actively engage in their self-care and therapeutic processes and with their supporting healthcare professionals.

**Figure 3:**
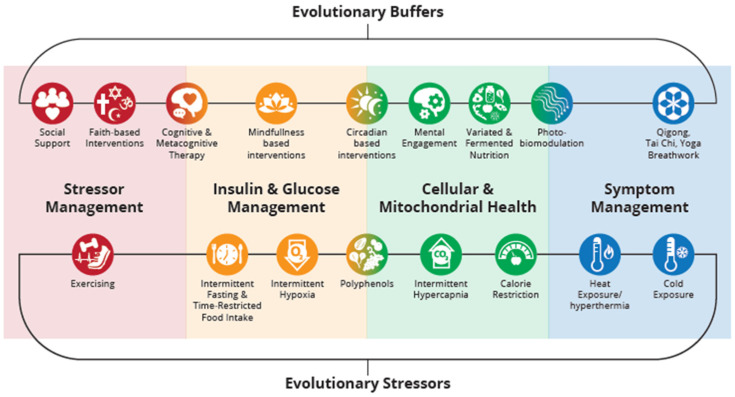
Evidence-based interventions within the mitochondrial information processing (MIPS) framework can enhance insulin resilience, a newly proposed health outcome resulting from the improvement of psychosocial factors and the implementation of evolutionary interventions. This ultimately contributes to positive health outcomes in conditions such as Type 2 Diabetes Mellitus (T2DM) with musculoskeletal (MS) problems and subsequent Chronic Non-Communicable Diseases (CNCDs), where the occurrence of one chronic condition increases the likelihood of developing additional diseases over time. Heart Rate Variability (HRV) is proposed as an additional parameter in the diagnostic toolbox, alongside existing parameters.

The proposed theoretical framework, as well as the interventions and measurement tools proposed, needs further validation, and we, therefore, recommend quality research to confirm the above. A full overview of our theoretical framework and subsequent interventions can be found in Figure 3.

## 4. Conclusions

This article proposed an expanded view of INS as a metabolic-socio-psychological substance within the MIPS framework, suggesting its roles beyond metabolism, particularly in cooperating with mitochondria in processing psychosocial factors into the biological fabric. By exploring INS’s potential involvement in mitochondrial sensing, integration, and transduction of psychosocial factors, we explored a theoretical framework that might link INS to a broader socio-psycho-biological approach to health.

Additionally, we adopted an evolutionary perspective, considering how modern psychosocial stressors may misalign with human evolutionary expectations. Mismatches between ancient evolutionary stressors (such as acute physical threats) and modern chronic stressors (such as social isolation or workplace pressures) might lead to chronic diseases, including T2DM with musculoskeletal and neurological complications.

Conversely, we proposed that integrative interventions aligned with evolutionary expectations—such as hormesis, intermittent fasting, physical exercise, breath work, and environmental stress exposure—might improve insulin resilience. Evolutionary buffers like diverse plant-based diets, fermentation, and maintaining circadian rhythms could also support metabolic and mitochondrial health.

Modern therapies, including photobiomodulation (PBM), but above all, psychosocial interventions such as mindfulness-based interventions, social network support and psychoeducation, meta-cognitive therapy and faith-based interventions, might offer integrative approaches to managing INS-implicated conditions. To evaluate these interventions, Heart Rate Variability (HRV) was proposed as a potential complementary diagnostic tool, alongside traditional clinical markers like HbA1c and insulin sensitivity indices. HRV’s sensitivity to psychosocial stress and metabolic changes suggests that it might be a useful, non-invasive method for tracking intervention outcomes, especially in patients with T2DM and related complications.

Ultimately, viewing INS as a social substance within the MIPS framework suggests that addressing psychosocial factors and evolutionary expectations might be essential in managing INS-related diseases. This comprehensive perspective could enhance our understanding of INS beyond its metabolic functions and promote a more integrative, evidence-based approach to chronic disease management. However, this theoretical framework and the proposed interventions need further empirical validation through clinical studies to assess their effectiveness and practical application.

By incorporating psychosocial, biological, and evolutionary components, future research might focus on testing the proposed interventions and frameworks, with the goal of creating more holistic approaches to managing T2DM and other INS-implicated conditions. Addressing upstream psychosocial needs, alongside downstream metabolic processes, might improve patient outcomes and treatment efficacy.

## Abbreviations

ACE	Adverse childhood events
AGEs	Advanced glycation end-products
AIF	Apoptosis-inducing factor
AKT	AKT-kinase (Protein Kinase B)
BAD	Bcl-2-associated death promoter
BAX	Bcl-2-associated X protein
BCL2	B-cell lymphoma 2
Ca^2+^	Calcium ions
CAMP	Cyclic adenosine monophosphate
CNCDs	Chronic non-communicable diseases
CORT	Cortisol
cf-mtDNA	Cell-free mitochondrial DNA
CREB	cAMP response element-binding protein
CYTC	Cytochrome c
DAG	Diacylglycerol
Drp1	Dynamin-related protein 1
DST	Dexamethasone suppression test
ECC	Excitation-contraction coupling
ECM	Extracellular matrix
ER	Endoplasmic reticulum
ERK1/2	Extracellular signal-regulated kinases 1 and 2
FBIs	Faith-based interventions
FM	Fibromyalgia
FoxO1	Forkhead box protein O1
FOS	FBJ murine osteosarcoma viral oncogene homolog
GDF15	Growth differentiation factor 15
GSK-3β	Glycogen synthase kinase 3 beta
HPA	Hypothalamus-pituitary-adrenal axis
HRV	Heart rate variability
IGF-1	Insulin-like growth factor 1
IGF1Rs	Insulin-like growth factor 1 receptors
INS	Insulin
INR	Insulin receptor
INSR/IGF1Rs	Hybrid insulin receptors/insulin-like growth factor 1 receptors
INSRs	Insulin receptors
INSS	Insulin signaling
IR	Insulin resistance
MAPK	Mitogen-Activated Protein Kinase
MAMs	Mitochondria-associated membranes
MIPS	Mitochondrial Information Processing System
MDPs	Mitochondrial-derived peptides
MIPs	Mitochondrial Information Processors
mTOR	Mechanistic target of rapamycin
mtDNA	Mitochondrial DNA
MOTS-c	Mitochondrial Open Reading Frame of the 12S rRNA-c
MOMP	Mitochondrial outer membrane permeabilization
MIMP	Mitochondrial inner membrane permeabilization
MSD	Musculoskeletal disorders
MSDs	Musculoskeletal disorders
MSK	Musculoskeletal
MSS	Musculoskeletal system
mTORC1	Mechanistic target of rapamycin complex 1
MYC	Myelocytomatosis oncogene
NAD(+)/NADH	Nicotinamide adenine dinucleotide (oxidized form)/Nicotinamide adenine dinucleotide (reduced form)
NF-kB	Nuclear factor kappa-light-chain-enhancer of activated B cells
OXPHOS	Oxidative phosphorylation
PI3K	Phosphoinositide 3-kinase
PGC-1α	Peroxisome proliferator-activated receptor gamma coactivator 1-alpha
PNI	Psychoneuroimmunology
RAS	Rat sarcoma
RAF	Rapidly Accelerated Fibrosarcoma kinase
RCT	Randomized Controlled Trial
ROS	Reactive oxygen species
SAM	Sympathetic-adrenomedullary axis
SHLPs	Small HN-like peptides
SM	Skeletal muscle
SR	Sarcoplasmic reticulum
T2DM	Type 2 Diabetes Mellitus
TCA	Tricarboxylic acid cycle
ULK1	Unc-51 like autophagy activating kinase 1
